# Effects of Bovine Milk-Derived Extracellular Vesicles on a 3D Intestinal Stromal Compartment

**DOI:** 10.3390/cells15030242

**Published:** 2026-01-27

**Authors:** Georgia Pennarossa, Sharon Arcuri, Madhusha Prasadani, Fulvio Gandolfi, Alireza Fazeli, Tiziana A. L. Brevini

**Affiliations:** 1 Laboratory of Biomedical Embryology and Tissue Engineering, Department of Veterinary Medicine and Animal Sciences, Centre for Stem Cell Research, Università degli Studi di Milano, 26900 Lodi, Italy; georgia.pennarossa@unimi.it (G.P.); sharon.arcuri@unimi.it (S.A.); 2 Institute of Veterinary Medicine and Animal Sciences, Estonian University of Life Sciences, 50303 Tartu, Estonia; madhusha.gamage@emu.ee (M.P.); alireza.fazeli@emu.ee (A.F.); 3 Department of Agricultural and Environmental Sciences-Production, Landscape, Agroenergy, Università degli Studi di Milano, 20134 Milan, Italy; fulvio.gandolfi@unimi.it; 4 Department of Pathophysiology, Institute of Biomedicine and Translational Medicine, University of Tartu, 50303 Tartu, Estonia; 5 Department of Clinical Sciences, The University of Sheffield Medical School, Sheffield S10 2RX, UK

**Keywords:** 3D stromal compartment, bovine milk-derived extracellular vesicles, decellularization, intestinal bio-scaffolds

## Abstract

**Highlights:**

**What are the main findings?**

- Bovine milk-derived extracellular vesicles (MEVs) stimulate intestinal stromal fibroblast proliferation in a dose-dependent manner.
- Pre-conditioning three dimensional (3D) decellularized intestinal scaffolds with MEVs further boosts fibroblast growth and enhance bio-scaffold repopulation compared with conventional culture systems.

**What are the implications of the main findings?**

- MEVs may play a significant role in intestinal tissue remodeling and repair by supporting stromal fibroblast function and gut homeostasis.
- MEVs may represent a noninvasive and readily available source of bioactive vesicles with potential application in the development of novel serum-free, chemically defined culture media for advanced 3D models and intestinal artificial organs.

**Abstract:**

Milk is an essential component of the diet. Among its diverse molecular constituents, it contains nanoscale entities, known as extracellular vesicles (EVs), which play a pivotal role in intercellular communication. In particular, milk-derived EVs (MEVs) influence intestinal homeostasis by mitigating inflammatory responses, modulating gut microbiota composition, and contributing to epithelial integrity preservation and restoration. Currently, there are no information regarding their impact on intestinal connective tissue. Here, we investigate bovine MEV effects on the porcine gut stromal compartment, exposing intestinal decellularized bio-scaffolds repopulated with primary intestinal stromal fibroblasts, to different MEV concentrations (10^6^, 10^8^, and 10^10^ particles/mL). We observed a dose-dependent effect of MEVs on stromal fibroblast proliferation rate at concentrations higher than 10^6^ particles/mL. In addition, when MEVs were used to pre-condition the decellularized intestinal bio-scaffolds prior to cell repopulation, fibroblast growth was further boosted. Overall, these findings suggest that MEVs may play a significant role in promoting tissue remodeling and repair. This activity appears particularly relevant for enhancing intestinal homeostasis and resilience, as stromal fibroblasts contribute to the maintenance of gut integrity, barrier function, and immune balance. Moreover, the data here presented suggests the possibility of using MEVs to develop serum-free, chemically defined culture media for the generation of advanced three-dimensional (3D) models and intestinal artificial organs.

## 1. Introduction

Milk is a fundamental component of the diet, providing essential macronutrients, vitamins, and minerals that are critical for growth, development, and overall health [1]. Beyond its well-established nutritional value, milk also contains a diverse array of bioactive molecules that contribute to the maintenance of physiological homeostasis and to the prevention of disease [2,3]. Among these components, extracellular vesicles (EVs)—nanoscale, membrane-bound particles naturally secreted by all cell types—have emerged as key mediators of intercellular communication. Indeed, EVs are known to exert their biological effects by transporting a wide variety of functional cargo, including proteins, lipids, messenger RNAs (mRNAs), and microRNAs, thereby influencing numerous physiological and pathological processes [4,5].

During the last years, particular attention has been directed toward milk-derived extracellular vesicles (MEVs), owing to their stability, biocompatibility, and potential therapeutic properties [6]. A growing body of evidence suggests that MEVs play a significant role in the regulation of intestinal homeostasis through several mechanisms [7]. Specifically, it has been demonstrated that MEVs and their labile cargo resist enzymatic degradation in the harsh conditions of the gastrointestinal tract and are selectively taken up by intestinal epithelial cells via endocytosis [8]. Once internalized, MEVs contribute to gut health by dampening intestinal inflammation, modulating immune responses, influencing the composition and activity of gut microbiota, and promoting the maintenance and repair of the epithelial barrier [7,9,10]. Despite this substantial knowledge, to date, research has primarily focused on the interactions between MEVs and the intestinal epithelium, whereas their effects on the underlying stromal compartment remain largely unexplored. Given the critical role of the stromal microenvironment in supporting epithelial function, mediating immune responses, and maintaining tissue architecture, understanding whether and how MEVs influence stromal cells is essential to fully elucidate their therapeutic potential in gut health and disease [11,12].

Traditional in vitro approaches used to study intestinal stromal biology have largely relied on two-dimensional (2D) culture systems, which fail to recapitulate the complex three-dimensional (3D) organization, biochemical composition, and mechanical properties of the native intestinal extracellular matrix (ECM). These features are known to critically regulate fibroblast behavior, including cell morphology, proliferation, mechanotransduction, and cell–matrix interactions. In this context, 3D models based on decellularized intestinal scaffolds represent a valuable tool, as they preserve tissue-specific ECM architecture and provide a biomimetic microenvironment that more closely resembles in vivo conditions, thereby enabling a more physiologically relevant assessment of fibroblast responses and improving the biological and translational relevance of in vitro findings.

Based on this, in the present study, we investigated the effects of bovine MEVs on a porcine 3D gut stromal compartment. To this end, we first generated porcine intestinal decellularized bio-scaffolds to create a 3D in vitro model of the gut stromal tissue that closely mimics the native ECM environment [13]. In parallel, we isolated and cultured primary stromal fibroblasts from the jejunum of adult pigs, which constitute the main cellular component of the intestinal connective tissue. Subsequently, we exposed intestinal decellularized bio-scaffolds repopulated with primary intestinal stromal fibroblasts to different MEV concentrations—10^6^, 10^8^, and 10^10^ particles/mL—in order to assess their potential effects on intestinal stromal cell proliferation.

## 2. Materials and Methods

All reagents were purchased from Thermo Fisher Scientific (Milan, Italy) unless otherwise indicated.

### 2.1. Ethical Statement

Intestinal tissues were collected at a local abattoir from adult swine. The organs were obtained from animals slaughtered for human consumption and therefore were not considered as part of animal experimentation under Directive 2010/63/EU of the European Parliament. All experiments were performed in accordance with the approved guidelines.

### 2.2. Collection of Porcine Intestines

**Figure 1:**
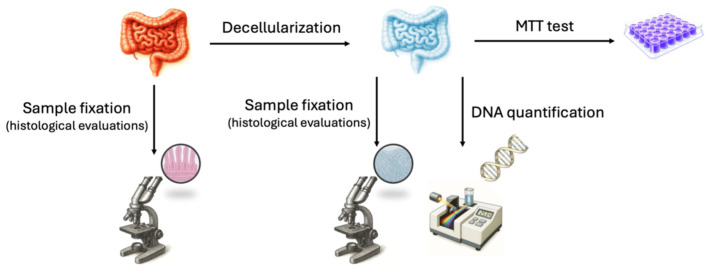
Schematic representation of the experimental workflow performed on intestinal tissue. MTT: 3-(4,5-dimethylthiazol-2-yl)-2,5-diphenyltetrazolium bromide.

Five intestinal samples (approximately 1.5 m each) from the jejunum of swine weighing approximately 120 kg were collected and transported to the laboratory in sterile cold phosphate-buffered saline (PBS, Sigma-Aldrich, Milan, Italy) supplemented with 2% antibiotic/antimycotic solution (Sigma-Aldrich, Milan, Italy). One intestinal segment (approximately 5 cm in length) from each animal was used to isolate porcine adult intestinal stromal cells. Another fragment of similar length was immediately fixed in 10% buffered formalin to serve as a native tissue control for histological evaluation. The remaining tissue samples were subjected to a decellularization protocol to generate intestinal bio-scaffolds, which were subsequently used for histological analyses, DNA quantification, and 3-(4,5-dimethylthiazol-2-yl)-2,5-diphenyltetrazolium bromide (MTT) assays, as outlined in Figure 1.

### 2.3. Isolation and Culture of Porcine Adult Intestinal Stromal Fibroblasts

Intestinal samples of 5 cm^2^ (around 2 g) were collected from the jejunum of three swine weighing approximately 120 kg, immersed in sterile PBS (Sigma-Aldrich, Milan, Italy) containing 2% antibiotic/antimycotic solution (Sigma-Aldrich, Milan, Italy) and transported to the laboratory. Tissues were extensively washed in sterile PBS (Sigma-Aldrich, Milan, Italy), cut in fragments of ~2 mm^3^, and transferred into 0.1% gelatin-coated (Sigma-Aldrich, Milan, Italy) 35 mm^2^ Petri dishes (Sarstedt, Milan, Italy). Droplets of Dulbecco’s modified eagle medium (DMEM) supplemented with 20% fetal bovine serum (FBS), 2 mM glutamine (Sigma-Aldrich, Milan, Italy), and 2% antibiotic/antimycotic solution (Sigma-Aldrich, Milan, Italy) were added onto each fragment and culture dishes were transferred in 5% CO_2_ incubator at 37 °C in humidified chambers. After 6 days, porcine intestinal fibroblasts started to grow out of the original fragments, and these were carefully removed. Cells were maintained in culture using the medium described above, in a 5% CO_2_ incubator at 37 °C and passaged twice a week at a 1:3 ratio. The primary porcine cell lines obtained from each animal were used in triplicate in 3 independent experiments.

### 2.4. Creation of Decellularized Intestinal Bio-Scaffolds

Upon arrival at the laboratory, jejunum samples were extensively washed in fresh sterile PBS, cut it into pieces of 10 cm in length and subjected to the decellularization protocol previously described by Arcuri et al. [13]. Briefly, intestinal fragments were frozen at −80 °C for at least 24 h, thawed at 37 °C in a water bath for 30 min, and transversally cut it into small pieces of around 5 cm in length. The intestinal mucosa and submucosal compartments were then mechanically dissociated from the tunica muscularis and serosa and incubated overnight in 1% Triton X-100 (Sigma-Aldrich, Milan, Italy) in deionized water (DI H_2_O). This step was followed by a 12 h wash in DI H_2_O and a subsequent treatment with 2% deoxycholate (Sigma-Aldrich, Milan, Italy) in DI H_2_O for 12 h. The obtained decellularized intestinal bio-scaffolds were extensively washed in DI H_2_O for 12 h and sterilized with 70% ethanol and 2% antibiotic/antimycotic solution in sterile H_2_O for 30 min. All the steps described above were performed at room temperature using an orbital shaker at 300 rpm.

### 2.5. Histological Analysis

Samples were fixed in 10% buffered formalin for 24 h at room temperature, dehydrated in graded alcohols, cleared with xylene, and embedded in paraffin. Serial microtome sections (5 μm thick) were cut, dewaxed, rehydrated and stained with hematoxylin and eosin (H&E, BioOptica, Milan, Italy), Crossmon’s trichrome, Gomori’s aldehyde-fuchsin (Bio-optica, Milan, Italy) or Alcian blue pH 2.5 (Bio-optica, Milan, Italy). Samples were analyzed with a Leica DMR microscope equipped with a Nikon DS-Ri2 camera (Nikon, Tokyo, Japan). Pictures were acquired with NIS-Elements D software, version 5.20 (Nikon, Tokyo, Japan). Native intestines were used as a control.

### 2.6. Stereological Analysis

The volume density (Vv) of collagen, elastin, and glycosaminoglycans (GAGs) was quantified using a well-established stereological point-counting method that allows the estimation of three-dimensional structural proportions from two-dimensional tissue sections, following Delesse’s principle. Tissue sections stained with Crossmon’s trichrome, Gomori’s aldehyde-fuchsin, and Alcian blue were randomly imaged. A point-count stereological grid with equally spaced test points was superimposed onto each image. Points intersecting the specific extracellular matrix component of interest (collagen, elastin, or GAGs) and points intersecting the total tissue area within the scaffold (used as the reference compartment) were counted. The relative volume density of each structure was calculated as the ratio between the number of points hitting the analyzed component of interest to the total points hitting the reference compartment, expressed as a percentage using the formula:

$$Vv\left(\mathrm{analyzed}\;\mathrm{compartment},\;\mathrm{reference}\;\mathrm{compartment}\right)\;=\frac{\sum P(\mathrm{analyzed}\;\mathrm{compartment})}{\sum P(\mathrm{reference}\;\mathrm{compartment})}\times 100$$

where ∑*P*(analyzed compartment) is the number of points falling on the compartment of interest, and ∑*P*(reference compartment) is the number of points falling on the reference structure. Three independent experiments were performed at least in triplicate.

### 2.7. Cell Density

Cell number was assessed by counting five 4′,6-diamidino-2-phenylindole (DAPI, Sigma-Aldrich, Milan, Italy) stained serial microtome sections obtained from each sample. Images were acquired from 5 randomly selected fields for each section and analyzed with ImageJ software version 1.53 (http://rsbweb.nih.gov/ij/index.html, accessed on 1 May 2023), following the provider’s instructions. Cell density was reported as the number of cells per mm^2^ of tissue. Three independent experiments were performed, each at least in triplicate.

### 2.8. Cytotoxicity Assessment

Intestinal bio-scaffold cytotoxicity was evaluated using the MTT assay. STO cells (CRL-1503, ATCC, Manassas, VA, USA) were seeded at 5 × 10^3^ cells/mL in 96-well plates. After 24 h, 20 mg of decellularized intestinal bio-scaffolds were added and co-cultured for 24, 48, and 72 h. Ten microliters of MTT solution were added and incubated for 4 h, after which formazan crystals were dissolved overnight in 100 μL of 10% SDS in 0.01 M hydrochloric acid (HCl). Optical density (OD) was measured at 550 nm. In the control group (CTR), 5 × 10^3^ STO cells/mL were seeded without the addition of bio-scaffolds. Three independent experiments were performed, each at least in triplicate.

### 2.9. MEV Production and Characterization

MEVs were provided by ConceptEasy OÜ (ConceptEasy OÜ, Tartu, Estonia). MEVs were isolated as described previously by Sapugahawatte et al. [14]. In brief, commercially available pasteurized low-fat cow milk (1.8% Tere joogipiim, TERE AS, Lelle 22, Tallinn 11318, Estonia) was used as the starting material for MEV enrichment. Milk was acidified to pH 4.6 using glacial acetic acid, followed by incubation at 4 °C for 1 h to induce casein coagulation. The resultant whey solution was subjected to sequential filtration using filter papers and 0.45 µm membrane filters. The filtrates were further processed by tangential flow filtration (TFF) using a benchtop TFF system (Centramate, Cytiva, MA, USA) equipped with a 300 kDa molecular weight cut-off polyethersulfone membrane (Omega PES, 0.02 m^2^ surface area, Cytiva, MA, USA), operated in a closed-loop configuration. The retentate was concentrated until the desired enrichment was achieved. The concentrate was further concentrated, and the buffer was exchanged to PBS (Sigma-Aldrich). Enriched MEVs were characterized in accordance with the guidelines of the International Society for Extracellular Vesicles (ISEV). MEV samples (120 μL at 10^12^ particles/mL), frozen at −80 °C, were subjected to a freeze dryer (Christ, Alpha 2-4 LDplus, Osterode am Harz, Germany) and lyophilized MEVs were stored in 4 °C until used for experiments. On the day of experiments, 120 μL PBS was used to reconstitute the lyophilized EV sample for further use.

MEVs were characterized by the manufacturer (Concepteasy OÜ, Tartu, Estonia) and characterization information was provided by the manufacturer (MEV datasheet is provided in the Supplementary Materials) [5,15,16,17,18].

### 2.10. Treatment of the 3D Artificial Stromal Compartment with MEVs

#### 2.10.1. Experimental Design

The effects of bovine MEVs on the porcine artificial gut stromal compartment were investigated by exposing the generated intestinal bio-scaffolds and intestinal stromal fibroblasts to different MEV concentrations (ConceptEasy, Tartu, Estonia): 10^6^, 10^8^, and 10^10^ particles/mL. MEVs were added in serum-free control medium (SFC) to avoid potential contamination by serum-derived EVs. As summarized in Table 1, the experimental groups were MEV pre-conditioned bio-scaffolds + untreated stromal cells; untreated bio-scaffolds + MEV conditioned stromal cells; MEV pre-conditioned bio-scaffolds + MEV conditioned stromal cells; untreated bio-scaffolds + untreated stromal cells cultured in standard culture medium supplemented with 10% FBS (CTR FBS); untreated bio-scaffolds + untreated stromal cells cultured in serum-free medium (SFC).

#### 2.10.2. Repopulation of Intestinal Bio-Scaffolds

1 × 10^6^ stromal fibroblasts/cm^2^ were seeded onto 3 × 3 × 3 mm^3^ intestinal bio-scaffold fragments and cultured in 5% CO_2_ at 37 °C for 6 days. Medium was refreshed every day. Cultures were arrested on days 1, 2, 4 and 6 for DNA quantification and histological evaluations.

#### 2.10.3. DNA Quantification

Five samples, weighing between 15 and 25 mg, were obtained from each experimental group. The weight of each fragment was recorded for subsequent DNA content analysis. Genomic DNA was isolated using the PureLink^®^ Genomic DNA Kit, following the manufacturer’s protocol. DNA concentrations were measured using the NanoDrop 8000 spectrophotometer (Thermofisher, Milan, Italy).

#### 2.10.4. Cell Doubling Time

Doubling time was calculated by counting nuclei in three serial microtome sections from each sample, stained with 4′,6-diamidino-2-phenylindole (DAPI; Sigma-Aldrich, Milan, Italy) at 24, 48, and 72 h. Cell numbers (*N*) at each time point were recorded, and doubling time was calculated using the following formula:

$$\mathrm{DT}=\frac{t\cdot ln2}{ln(N_{t}/N_{0})}$$

where

- $N_{0}$ = initial number of cells at time 0;
- $N_{t}$ = number of cells at time t (hours);
- t = elapsed time between measurements (hours).

For each experimental group, including CTR FBS, SFC, and MEV-treated cells at different concentrations (10^6^, 10^8^, 10^10^ particles/mL), doubling time was calculated for each replicate and expressed as mean ± standard deviation (SD) from three independent experiments.

### 2.11. Statistical Analysis

Statistical analysis was performed using one-way ANOVA (SPSS 19.1; IBM). Data were presented as mean ± standard deviation (SD). Differences of *p* ≤ 0.05 were considered significant and were indicated with different superscripts.

## 3. Results

### 3.1. Creation and Characterizatiojn of Decellularized Intestinal Bio-Scaffolds

**Figure 2:**
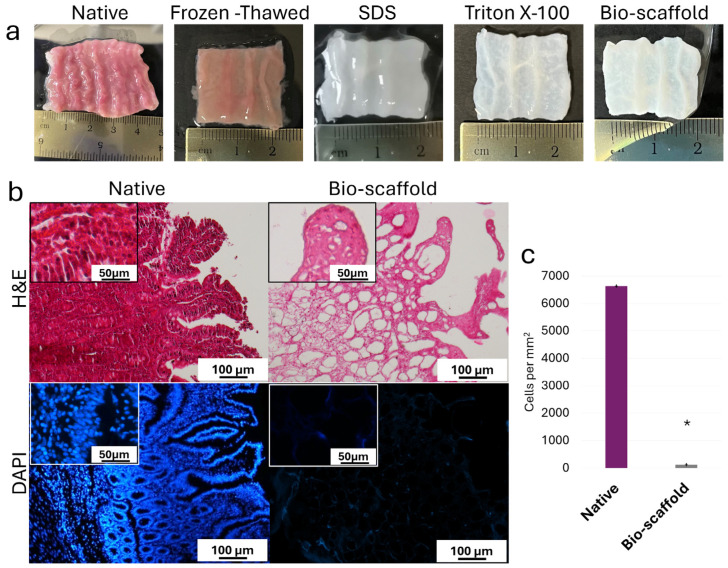
**Macroscopic and microscopic evaluations of native intestinal tissues (Native) and decellularized intestine (Bio-scaffold).** (**a**) Chronological macroscopic images illustrating the decellularization process. Native intestine and bio-scaffolds display comparable morphology, while the color turns from red (Native) to white (Bio-scaffold). (**b**) Representative image of H&E staining shows fewer nuclei in the decellularized bio-scaffolds than the untreated tissue (Native). DAPI staining displays the presence of nuclei in the native tissue and their disappearance after the decellularization process (Bio-scaffold). (**c**) Cell density. A lower number of nuclei were observed in the decellularized bio-scaffolds than the untreated tissues (Native). Data are expressed as the mean ± standard deviation (SD), * *p*  <  0.05.

During the decellularization process, intestinal tissues maintained their typical morphology with visible villi and retained shape of the native tissue, without any deformation (Figure 2a). In parallel, the color turned from red to white (Figure 2a). H&E staining demonstrated the absence of basophilic staining in the generated intestinal bio-scaffolds (Bio-scaffold, Figure 2b), while both basophilic and eosinophilic staining were visible in native untreated tissue (Native, Figure 2b). DAPI staining (Figure 2b) and cell density analyses (Figure 2c) confirmed a significantly lower number of nuclei compared with untreated tissues (Native).

**Figure 3:**
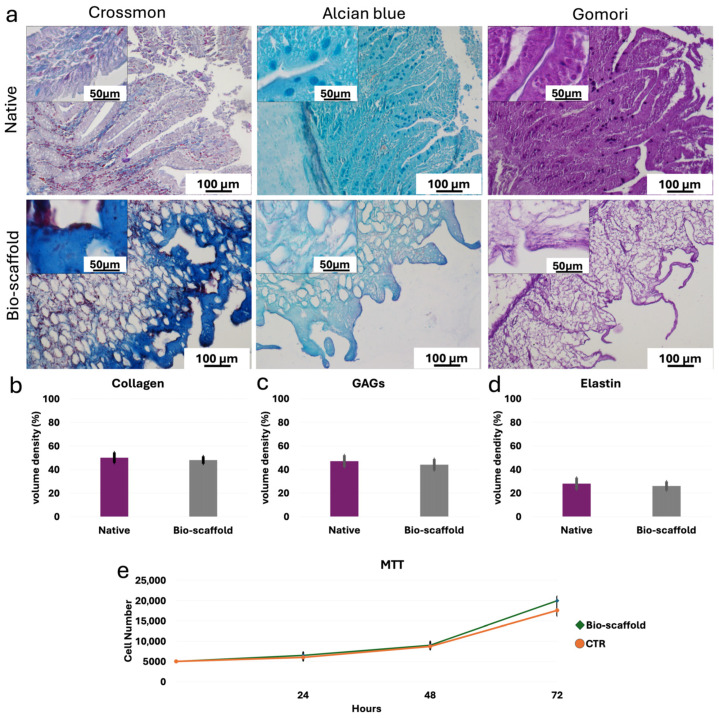
**ECM microarchitecture and composition of native intestinal tissues (Native) and decellularized intestine (Bio-scaffold).** (**a**) Crossmon’s trichrome, Alcian blue and Gomori’s aldehyde-fuchsin staining show the preservation of collagen fibers (blue), GAGs (light blue) and elastin (magenta), respectively, as well as their comparable distribution between Native tissues (upper panels) and intestinal Bio-scaffolds (lower panels). (**b**) Collagen stereological analysis demonstrates no significant differences between Native and Bio-scaffold groups. Data are expressed as the mean ± standard deviation (SD). (**c**) GAG quantifications indicate no significant differences between Bio-scaffolds and Native tissues. Data are expressed as the mean ± standard deviation (SD). (**d**) Elastic fiber analysis shows comparable amount before (Native) and after the decellularization process (Bio-scaffold). Data are expressed as the mean ± standard deviation (SD). (**e**) MTT assay demonstrates no significant differences between cells co-cultured with the generated bio-scaffolds and those of the control (CTR) at the different time points analyzed. Data are expressed as the mean ± standard deviation (SD).

Histochemical assessments demonstrated the preservation of the three ECM components analyzed at the end of the decellularization process. In particular, Crossmon’s trichrome staining showed the maintenance of collagen fibers with a comparable distribution between native tissues and bio-scaffolds (Figure 3a). The morphological observations were confirmed by stereological analyses, where no significant differences were detected between native tissues and decellularized bio-scaffolds (Figure 3b). Alcian blue staining and their measurements revealed GAG retention in the intestinal bio-scaffolds, when compared to the native tissues (Figure 3a,c). Gomori’s aldehyde-fuchsin staining (Figure 3a) and their quantifications (Figure 3d) indicated the maintenance of elastic fibers at the end of the decellularization process.

In addition, MTT assay demonstrated no cytotoxic effects exerted by the generated intestinal bio-scaffolds. In particular, no significant differences in cell growth were detected between cells co-cultured with the intestinal bio-scaffolds and those of the control group (CTR; Figure 3e).

### 3.2. Effects of MEVs on the Stromal Compartment of 3D Artificial Intestines

**Figure 4:**
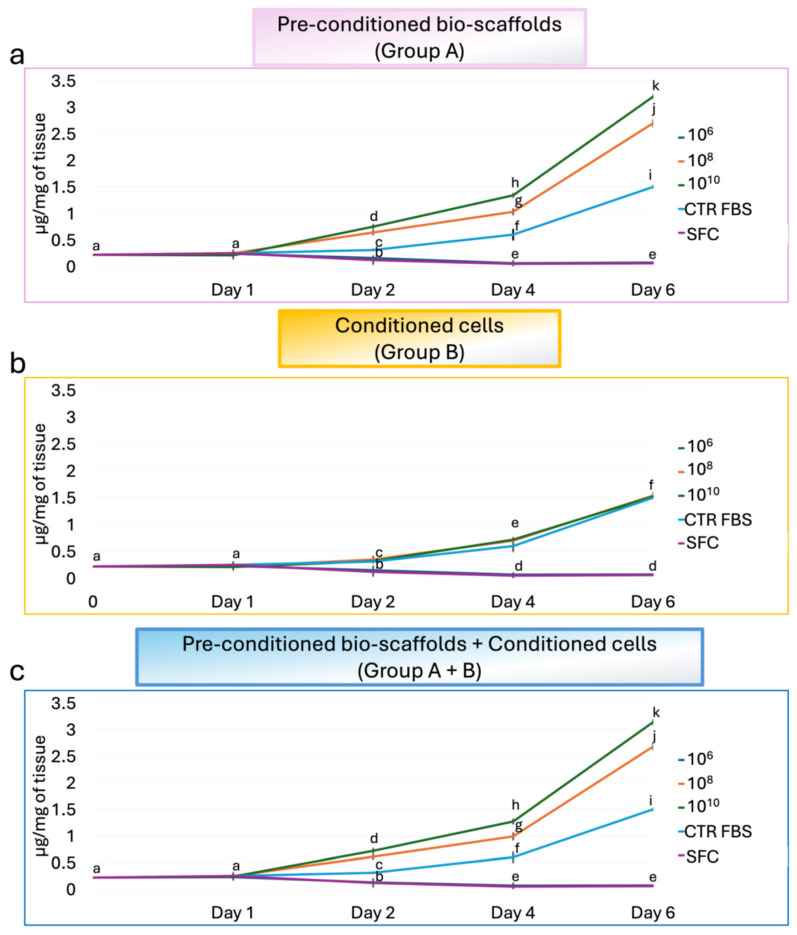
**Effects of MEVs on intestinal stromal cell proliferation.** DNA quantification of intestinal stromal cells cultured on decellularized intestinal bio-scaffolds at days 1, 2, 4, and 6. (**a**) DNA content measurement demonstrated that a pre-conditioning intestinal bio-scaffolds (Group A) with 10^8^ and 10^10^ MEV particles/mL induced a statistically significant increase in the proliferation of engrafted stromal cells in a dose-dependent manner, with values significantly higher than those observed in the CTR FBS group. The lowest concentration of 10^6^ MEV particles/mL had no effect, and values were comparable to the SFC group. Different lowercase letters indicate statistically significant differences between groups at the same time point (*p* < 0.05). Data are expressed as mean ± standard deviation (SD). (**b**) DNA quantification indicated that direct exposure of stromal cells (Group B) to 10^8^ and 10^10^ MEV particles/mL resulted in proliferation rates similar to those observed in the CTR FBS group, regardless of the dose used, while 10^6^ MEV particles/mL had no effect, and values were comparable to the SFC group. Different lowercase letters indicate statistically significant differences between groups at the same time point (*p* < 0.05). Data are expressed as mean ± standard deviation (SD). (**c**) Total DNA content analysis showed that the simultaneous use of 10^8^ and 10^10^ MEV particles/mL to pre-condition the intestinal bio-scaffold and to treat stromal cells (Group A+B) induced a statistically significant increment in the proliferation of engrafted stromal cells in a dose-dependent manner, with values significantly higher than those observed in the CTR FBS group. In contrast, the values obtained using 10^6^ MEV particles/mL were comparable to the SFC group. Different lowercase letters refer to a statistically significant differences among groups at the same time point (*p* < 0.05). Data are expressed as mean ± standard deviation (SD).

DNA quantification indicated that MEVs exert a dose-dependent effect on stromal fibroblast proliferation (Figure 4). Specifically, the lowest concentration of 10^6^ MEV particles/mL had no effect on fibroblast proliferation, with values comparable to the SFC group, regardless of the treatment applied (Figure 4a–c). In contrast, direct exposure of stromal cells to 10^8^ and 10^10^ MEV particles/mL (Group B), resulted in proliferation rates similar to those observed in the CTR FBS group, independent of the dose (Figure 4b). Notably, pre-conditioning intestinal bio-scaffolds with 10^8^ and 10^10^ MEV particles/mL (Group A) significantly enhanced the proliferation of engrafted stromal cells in a dose-dependent manner, with values significantly higher than those observed in the CTR FBS group (Figure 4a). When MEVs were used both to pre-condition the intestinal bio-scaffold and to treat stromal cells simultaneously (Group A+B), proliferation rates remained comparable to those in Group A (Figure 4c).

**Figure 5:**
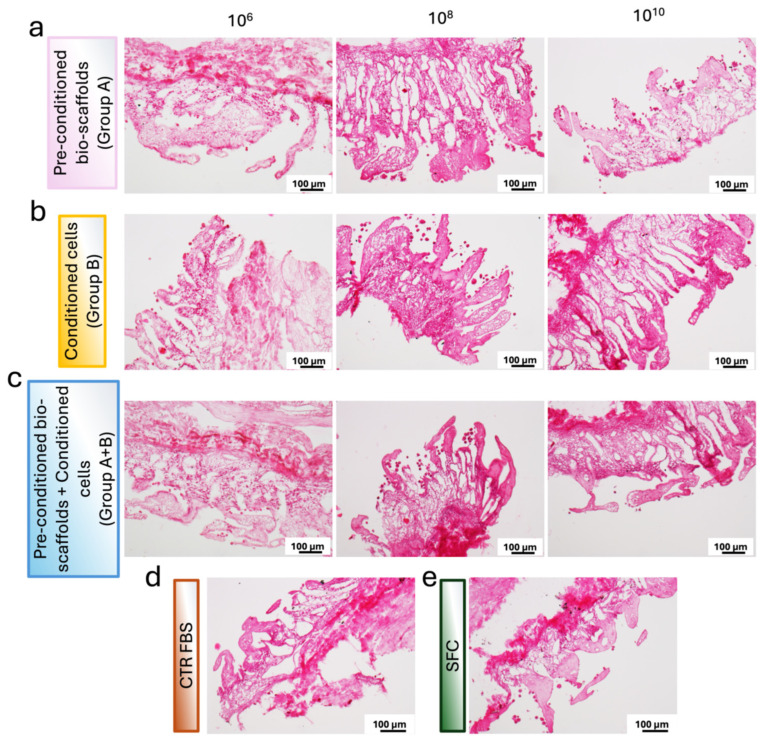
**Representative images of H&E-stained sections of repopulated bio-scaffolds.** (**a**) Pre-conditioned bio-scaffolds (Group A). (**b**) MEV-conditioned stromal cells seeded onto untreated bio-scaffolds (Group B). (**c**) MEV-conditioned stromal cells seeded onto pre-conditioned bio-scaffolds (Group A+B). (**d**) Stromal cells cultured in serum-supplemented standard medium (CTR FBS). (**e**) Stromal cells cultured in serum-free medium (SFC). Differences in cell distribution and tissue colonization are evident among the experimental groups.

These findings were supported by histological analyses. In more detail, H&E staining revealed an even distribution of stromal cells throughout the scaffold, confirming effective cell engraftment and preservation of tissue architecture across all experimental conditions (Figure 5). In addition, samples from Group A and Group A+B exhibited similarly dense and well-organized cellular networks, indicating comparable levels of cell expansion between these two conditions. The cellular density observed in both Group A and Group A+B was, indeed, higher than that detected in Group B. These morphological observations are in line with the quantitative DNA measurements, which showed increased proliferation rates in Group A and Group A+B compared with Group B.

**Figure 6:**
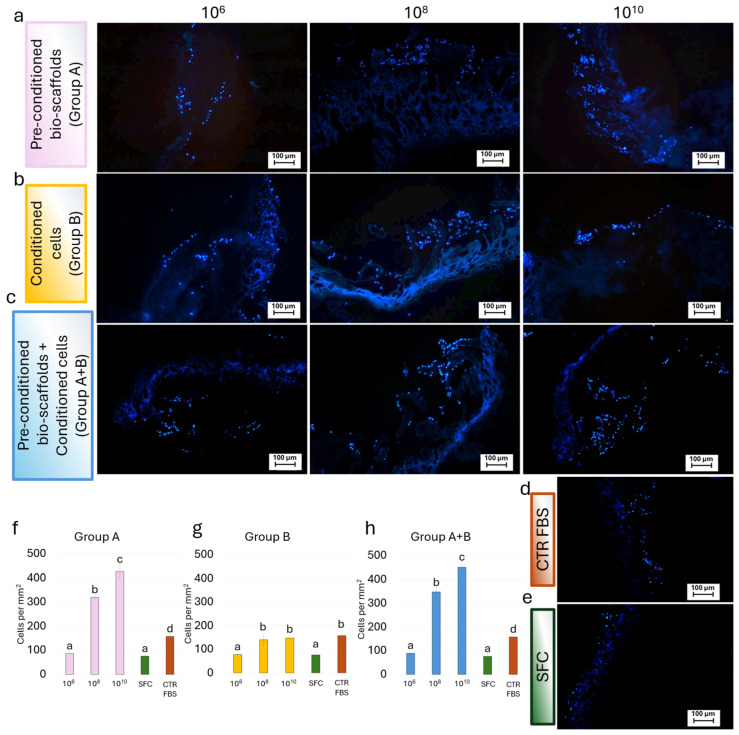
**Representative fluorescence images of DAPI-stained repopulated bio-scaffolds.** Blue nuclear staining indicated cell localization and distribution within the scaffold structure for (**a**) Pre-conditioned bio-scaffolds (Group A); (**b**) MEV-conditioned stromal cells seeded onto untreated bio-scaffolds (Group B); (**c**) MEV-conditioned stromal cells seeded onto pre-conditioned bio-scaffolds (Group A+B); (**d**) Stromal cells cultured in serum-supplemented standard medium (CTR FBS); and (**e**) Stromal cells cultured in serum-free medium (SFC). (**f**) Cell density analysis showed that a pre-conditioning intestinal bio-scaffolds (Group A) with 10^8^ and 10^10^ MEV particles/mL induced a statistically significant increment of cell nuclei in a dose-dependent manner, with values significantly higher than those observed in the SFC and CTR FBS groups. The values observed at the lowest concentration of 10^6^ MEV particles/mL were comparable to the SFC group. Different lowercase letters refer to statistically significant differences among groups at the same time point (*p* < 0.05). Data are expressed as mean ± standard deviation (SD). (**g**) Cell density analysis revealed that direct exposure of stromal cells (Group B) to 10^8^ and 10^10^ MEV particles/mL resulted in values comparable to those observed in the CTR FBS group. In contrast, treatment with 10^6^ MEV particles/mL did not induce any effect, yielding cell density comparable to those of the CTR w/o FBS group. Different lowercase letters refer to statistically significant differences among groups at the same time point (*p* < 0.05). Data are expressed as mean ± standard deviation (SD). (**h**) Cell density analysis showed that the simultaneous application of 10^8^ and 10^10^ MEV particles/mL, used both to pre-condition the intestinal bio-scaffold and to treat stromal cells (Group A+B), resulted in a statistically significant increase in the proliferation of engrafted stromal cells in a dose-dependent manner, with values significantly higher than those observed in the CTR FBS group. Treatment with 10^6^ MEV particles/mL did not enhance proliferation, yielding cell density values comparable to those of the SFC group. Different lowercase letters refer to a statistically significant differences among groups at the same time point (*p* < 0.05). Data are expressed as mean ± standard deviation (SD).

Similarly, analysis of DAPI-stained samples confirmed the widespread presence of cell nuclei throughout the bio-scaffolds in all experimental groups. These results mirrored the trends observed in H&E staining and DNA quantification, with Group A (Figure 6a,f) and Group A+B (Figure 6c,h) displaying higher cellular density compared to Group B (Figure 6b,g).

Consistently, the calculated doubling times of stromal cells corroborated these observations, with Groups A and A+B exhibiting shorter doubling times, whereas Group B showed longer doubling times comparable to the serum-free control (SFC) (Table 2).

**Figure 7:**
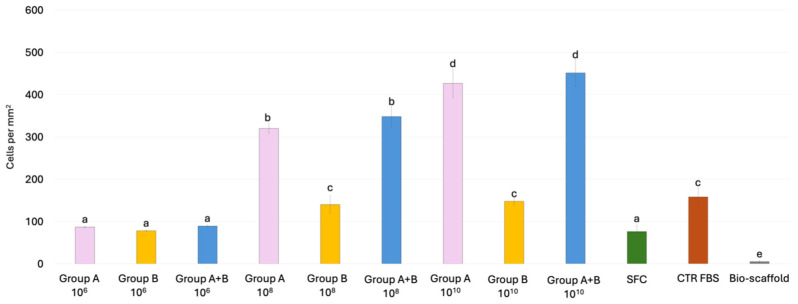
**Cell density analysis of intestinal bio-scaffolds repopulated with stromal cells under different experimental conditions.** Histogram showing the number of cell nuclei detected after 6 days of culture in Group A, Group B, and Group A+B, CTR FBS, SFC and Bio-scaffold. Data are expressed as the mean ± standard deviation (SD) (*p*  >  0.05). Different lowercase letters refer to statistically significant differences among groups at the same point (*p* < 0.05).

Cell density measurements demonstrate that the number of nuclei in the SFC group and the 10^6^ MEV particles/mL groups was comparable, irrespective of the treatment applied (Figure 7). In contrast, when the same MEV concentrations were directly added to stromal cells (Group B), the number of nuclei after 6 days of culture was similar to that of the CTR FBS group. In addition, pre-conditioning of bio-scaffolds with 10^8^ and 10^10^ MEV particles/mL (Group A) promoted dose-dependent proliferation of engrafted cells, with significantly higher cell densities than in the CTR FBS group. Finally, when 10^8^ and 10^10^ MEVs were applied both to pre-condition the scaffold and to treat stromal cells (Group A+B), nuclei counts were comparable to those observed in Group A (Figure 7).

## 4. Discussion

In the present study, we investigate the effects of bovine MEVs on the intestinal stromal compartment. To this purpose, we first generated decellularized intestinal bio-scaffolds that maintained the main structural and biochemical properties of the native tissue. The decellularization protocol described herein was shown to preserve the general morphology and villus architecture of the native intestine, demonstrating its effectiveness in maintaining ECM structural integrity. In agreement with our previous report [13], macroscopic observations showed tissue color turning from red to white, suggesting the removal of the cellular compartment that was also confirmed through H&E staining. This is consistent with DAPI immunofluorescence and quantitative measurement results that indicated a statistically significant depletion of nuclear material, thus validating the efficacy of the decellularization process used. These findings are in line with previous studies demonstrating that optimized decellularization protocols can generate acellular bio-scaffolds, while maintaining intact native tissue architecture and biochemical composition [19,20,21,22]. Furthermore, the data here reported are in agreement with previous evidence indicating that protocols combining detergents and enzymatic treatments can effectively remove cellular components from the intestinal tissue, while minimizing damage to ECM proteins and its microstructures. In particular, detergent-enzymatic treatments have been previously shown to preserve porcine ECM [23,24] and human intestinal tissues [25,26], respectively. Indeed, matrix preservation represents a critical aspect in the decellularization process, since ECM provides not only the mechanical cues but also essential biochemical signals that regulate cell behavior, including adhesion, migration, proliferation, and differentiation [27,28,29,30,31]. As evidenced by the present results, collagen fibers, essential for providing mechanical strength and scaffolding support, together with GAGs, which play a crucial role in hydration, signaling, and cell–matrix interactions, were well-preserved. A comparable trend was detected in elastic fibers that are responsible for tissue elasticity and resilience of the intestinal wall. These findings align well with previous studies carried out in the rat, where small bowel decellularization demonstrated preservation of connective tissue components, following a detergent-enzymatic treatment [19]. Similarly, a recent work on porcine large intestinal scaffolds reported successful maintenance of collagen and elastin content after perfusion decellularization, although accompanied by a reduction in GAG content, attributed to leaching during perfusion and freeze–thaw storage [32].

A crucial aspect that must be rigorously controlled is the persistence of residual detergents within decellularized ECM scaffolds, as their presence can compromise scaffold biocompatibility—both in vitro and in vivo—and negatively impact subsequent recellularization [33]. In the results reported here, MTT assays demonstrated no cytotoxic effects exerted by the obtained intestinal bio-scaffolds, thus indicating the effective removal of detergent residues and highlighting the suitability of the prepared bio-scaffolds for in vitro modeling and for broader applications in tissue engineering and regenerative medicine [34]. Recent advances in EV production, storage, and scale-up have significantly improved the accessibility and translational potential of EV-based technologies. Commercially produced EVs are now widely available and suitable for experimental and translational use. In the current study, we used EVs obtained from a commercial supplier. Importantly, the same EV preparations have been used and characterized by multiple independent laboratories, supporting their reproducibility, stability, and functional integrity [14,35]. Previous studies demonstrate that these bulk-produced EVs retain their key molecular and proteomic characteristics and remain functional following lyophilization. The availability of well-characterized, commercially produced EVs reduces the need for specialized in-house production and facilitates broader adoption, reproducibility, and translation of EV-based applications.

Based on this, we employed the decellularized bio-scaffold to examine how MEVs influence intestinal stromal cell behavior in vitro. Our results demonstrated that low-dose MEVs exerted no detectable effects on fibroblast proliferation. This is in agreement with earlier findings in intestinal epithelial cells cultured in 2D systems [36,37,38] and suggests that MEV concentrations higher than 10^6^ particles/mL are required to elicit cellular responses, both when cells are grown in traditional 2D culture conditions, as well as in more complex 3D environments [39,40,41]. As a matter of fact, measurable responses were observed when higher doses were used. In particular, direct addition of 10^8^ and 10^10^ MEV particles/mL to stromal cells promoted proliferation to levels comparable to those observed in serum-supplemented controls (CTR FBS group). It should be noted that FBS is known to contain EVs, although significant variability among different lots has been described in the literature. Specifically, untreated FBS-supplemented complete medium has been reported to contain on the order of ~10^10^ EVs/mL [42]. However, the proliferative effects observed in the experiments here described cannot be attributed to serum-derived EVs but rather to MEVs, since all culture conditions involving MEVs were conducted in the absence of FBS. Accordingly, a SFC group was included to control for serum deprivation, whereas the CTR FBS group was used as a standard reference condition, commonly employed in cell culture. Consistent with our results, EV-mediated stimulation of fibroblast or stromal cell proliferation has been previously reported in other tissues, including dermal and cardiac fibroblasts and mesenchymal stromal cells of human and murine origin, where EVs derived from stem or progenitor cells induced cell growth, migration, and tissue regeneration [43,44,45,46,47]. The data reported here further extend this concept to the intestinal microenvironment, providing the first evidence that MEVs can enhance 3D cultured intestinal fibroblast proliferation and suggesting that fibroblast targeted EV signaling may be a general mechanism across multiple tissues and different species. Interestingly, MEV-induced proliferative effects on stromal cells were particularly evident when 10^8^ and 10^10^ particles/mL were used to pre-treat the decellularized intestinal bio-scaffolds. Under these conditions, cell responses were significantly higher than those observed in FBS-supplemented controls (CTR FBS group) and remained stable even when MEVs were further added to engrafted stromal cells (Group A+B). Altogether these results indicate a general trophic effect exerted by MEVs on intestinal fibroblasts and suggest a synergistic interaction with ECM components to enhance stromal cell proliferation within a biomimetic 3D environment.

## 5. Conclusions

The data obtained in this study demonstrate MEV’s ability to enhance intestinal stromal fibroblast proliferation activity, a function that may contribute to the maintenance of gut barrier integrity and mucosal repair following injury or stress. These findings highlight new opportunities for the application of MEVs in nutraceutical, biomedical, and health-promoting formulations, where controlled modulation of fibroblast function and stromal–epithelial interactions could support gastrointestinal well-being and systemic homeostasis. Furthermore, the results reported herein suggest the possibility of using MEVs to develop serum-free, chemically defined culture media suitable for the generation of advanced 3D intestinal models and artificial organs, which may provide physiologically relevant platforms for regenerative medicine, disease modeling, and drug discovery.

## Figures and Tables

**Table 1:** Experimental groups for assessing MEV effects on the intestinal stromal compartment.

	Bio-Scaffolds	Stromal Fibroblasts	FBS
Pre-conditioned bio-scaffolds (Group A)	10^6^ MEVs	-	-
	10^8^ MEVs	-	-
	10^10^ MEVs	-	-
Conditioned cells (Group B)	-	10^6^ MEVs	-
	-	10^8^ MEVs	-
	-	10^10^ MEVs	-
Pre-conditioned bio-scaffolds + Conditioned cells (Group A+B)	10^6^ MEVs	10^6^ MEVs	-
	10^8^ MEVs	10^8^ MEVs	-
	10^10^ MEVs	10^10^ MEVs	-
CTR FBS	-	-	10%
SFC	-	-	-

**Table 2:** **Doubling time of intestinal stromal cells under different experimental conditions.** Data are expressed as mean ± standard deviation (SD) from three independent experiments.

Experimental Group		Mean Doubling Time (Hours)	SD (*n* = 3)
Pre-conditioned bio-scaffolds (Group A)	10^6^ MEVs	67.5	1.1
	10^8^ MEVs	26.3	0.5
	10^10^ MEVs	20.4	0.7
Conditioned cells (Group B)	10^6^ MEVs	67.2	1.0
	10^8^ MEVs	36.2	1.3
	10^10^ MEVs	36.6	1.1
Pre-conditioned bio-scaffolds + Conditioned cells (Group A+B)	10^6^ MEVs	67.9	0.9
	10^8^ MEVs	26.5	0.8
	10^10^ MEVs	21.0	0.4
CTR FBS		36.5	1.2
SFC		68.1	0.8

## Data Availability

The raw data supporting the conclusions of this article will be made available by the authors on request. The data are not publicly available due to privacy constraints, including data protection requirements associated with the project. Requests for data access will be evaluated on a case-by-case basis and subjected to appropriate approvals.

## Supplementary Materials

The following supporting information can be downloaded at https://www.mdpi.com/article/10.3390/cells15030242/s1: Figure S1: Characterization of milk EVs using NTA; Figure S2: Biochemical characterization of reconstituted lyophilized milk EVs using western blotting [5,15,16,17,18].
