# Supplementary material for: Effects of Bovine Milk-Derived Extracellular Vesicles on a 3D Intestinal Stromal Compartment
Source: Cells. 2026 Jan 27;15(3):242. doi: 10.3390/cells15030242 (PMC12896473; doi:10.3390/cells15030242)
Supplement: Supplementary file 1 [file cells-15-00242-s001.zip › cells-4041206-supplementary.pdf]

Cow’s Milk Extracellular vesicles

CONCEPTEASY
To Be Sure

About Extracellular vesicles

Extracellular vesicles (EVs) are membrane-bound nanoparticles carrying complex and heterogeneous cargo [5, 15-17]. EVs are naturally released from most cell types and can be observed in various biological fluids and cell culture-conditioned medium [16,17]. They play a vital role in intercellular communication and are involved in various physiological and pathological processes [15,17]. A number of factors, such as the cell type, physiological state, and environmental conditions, can affect how EV cargo is packaged and modified [5,17]. EVs cargo can reflect the originating cell’s characteristics and provide valuable information about health and function [5,15]. Research on EVs has gained significant attention in different fields such as cancer biology, neuroscience, immunology, reproduction, and regenerative medicine [15,17]. EVs potential applications are vast and include (but not limited) to diagnostic biomarkers, therapeutics, drug delivery systems, tools for understanding intercellular communication and disease processes [15,18].

Cow’s Milk Extracellular Vesicles

Concepteasy's purified cow’s milk EVs are isolated from cow’s milk using a combination of precipitation and tangential flow filtration (TFF) steps. Isolated EVs particle concentration and size distributions is measured using Nanoparticle Tracking Analysis (NTA). The total EV protein concentration is quantified using Bradford Protein Assay. The milk EVs are lyophilized for storage and transport. Lyophilized EVs after reconstitution, show similar physical characteristics similar to their physical characteristics before lyophilization.

Steps for reconstitution of lyophilized milk EVs

Milk lyophilized EVs can be reconstituted by adding 120 µL Phosphate-Buffered Saline (PBS) without calcium and magnesium to obtain a final EV concentration of $1x10^{12}$ particles/mL. Different volumes of the diluent buffer for EVs reconstitution can be chosen by the users in according with the desired final concentration.

i. Gently resuspend EVs by pipetting the solution without creating any air bubbles
ii. Vortex the reconstituted EVs for 30-60 seconds
iii. Centrifuge the reconstituted EVs for a short time and pipette the solution without introducing air bubbles. Upon completion of this step, the EVs are ready for use.

Storage of lyophilized and reconstituted EVs

Lyophilized milk EVs can be stored at 4°C for 24 months. Reconstituted milk EVs should be used within two hours of reconstitution. It is necessary to aliquot the remaining reconstituted EV sample and store it at -80°C for up to three months. It is advisable to avoid repeated freezing and thawing cycles.

Physical and biochemical characteristics of milk EVs

Physical characterization of milk EVs using NTA detected heterogeneous particle size distributions (Figure S1).

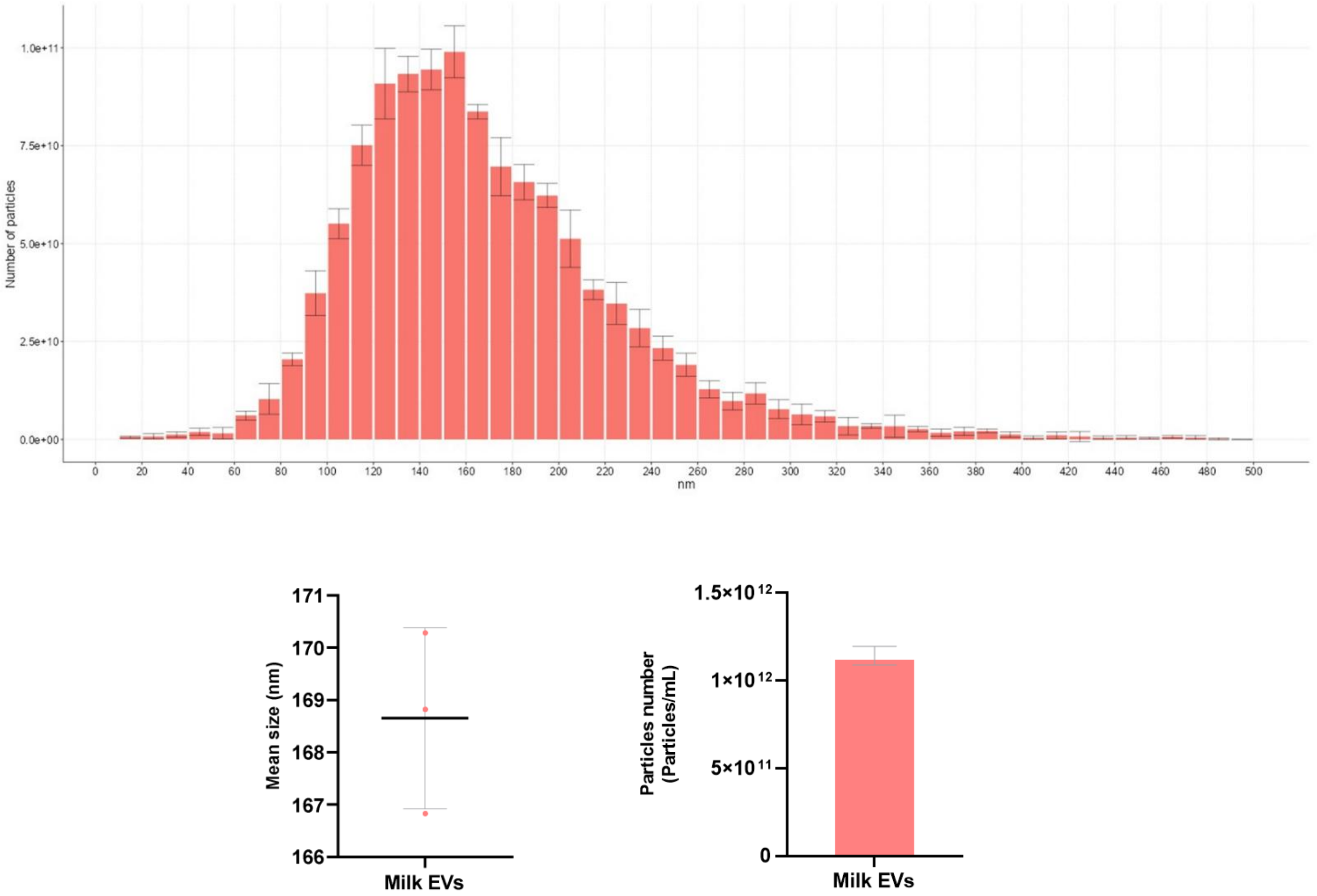

Figure S1. Characterization of milk EVs using NTA. The particle size distribution and concentration of reconstituted lyophilized milk EVs determined by NTA. Concentration of EVs are displayed as particles/mL (mean ± SD).

Western blot analysis confirmed that EV surface marker proteins were present in milk EVs (Figure S2).

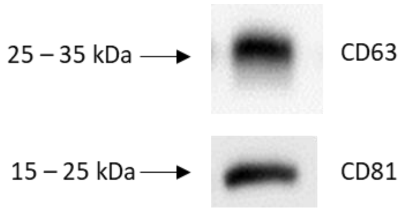

Figure S2. Biochemical characterization of reconstituted lyophilized milk EVs using western blotting. Western blot analysis detected the presence of EV tetraspanin protein markers in milk EVs (such as CD63 and CD81).
